# Subcellular Singlet Oxygen and Cell Death: Location Matters

**DOI:** 10.3389/fchem.2020.592941

**Published:** 2020-11-17

**Authors:** Pingping Liang, Dmytro Kolodieznyi, Yehuda Creeger, Byron Ballou, Marcel P. Bruchez

**Affiliations:** ^1^Molecular Biosensor and Imaging Center, Carnegie Mellon University, Pittsburgh, PA, United States; ^2^Department of Chemistry, Carnegie Mellon University, Pittsburgh, PA, United States; ^3^Key Laboratory of Flexible Electronics (KLOFE), Institute of Advanced Materials (IAM), Nanjing Tech University, Nanjing, China; ^4^Department of Biological Sciences, Carnegie Mellon University, Pittsburgh, PA, United States

**Keywords:** singlet oxygen, photoablation and photodynamic therapy, subcellular location, near-infrared, localized reactive oxygen species (ROS), chemoptogenetic, photosensitizer, fluorogen

## Abstract

We developed a tool for targeted generation of singlet oxygen using light activation of a genetically encoded fluorogen-activating protein complexed with a unique dye molecule that becomes a potent photosensitizer upon interaction with the protein. By targeting the protein receptor to activate this dye in distinct subcellular locations at consistent per-cell concentrations, we investigated the impact of localized production of singlet oxygen on induction of cell death. We analyzed light dose-dependent cytotoxic response and characterized the apoptotic vs. necrotic cell death as a function of subcellular location, including the nucleus, the cytosol, the endoplasmic reticulum, the mitochondria, and the membrane. We find that different subcellular origins of singlet oxygen have different potencies in cytotoxic response and the pathways of cell death, and we observed that CT26 and HEK293 cell lines are differentially sensitive to mitochondrially localized singlet oxygen stresses. This work provides new insight into the function of type II reactive oxygen generating photosensitizing processes in inducing targeted cell death and raises interesting mechanistic questions about tolerance and survival mechanisms in studies of oxidative stress in clonal cell populations.

## Introduction

Singlet oxygen (^1^O_2_) is the lowest electronic excited state of molecular oxygen that can be generated by biochemical, photochemical, and chemical processes. Unlike ground-state ^3^O_2_, ^1^O_2_ is a potent and reactive dienophile, natively involved in a variety of biological processes and biomedical technologies (Kessel, 2019b). The important distinction between type I (oxygen radical) and type II (singlet oxygen) reactions, and the importance of type II reactions in photochemistry, was clearly established by Foote (Foote, 1991; Greer, 2006); subsequent work has confirmed this [examples: (Doria et al., 2013; Ashen-Garry and Selke, 2014; Baptista et al., 2017)]. Singlet oxygen production by photosensitizers (PS) has been used for photoactive antiviral drugs (Vigant et al., 2013), for induction of apoptosis or necrosis (Bauer, 2016; Kessel, 2019a), and for generation of cytotoxic or immune-inducing signals in photodynamic tumor ablation (Turan et al., 2016; Kobayashi et al., 2020). The drawbacks of traditional PSs [e.g. porphyrins, chlorin e6, and methylene blue, are low water solubility (Yu et al., 2015) and no (or limited control of) target or cell-type selectivity (Kim et al., 2018) and reactive oxygen species selectivity, resulting in off-target cytotoxicity (Castaneda-Gill et al., 2017)]. Thus, we require water-soluble ^1^O_2_-specific PSs with stringent targeting and activation properties to improve the selectivity and impact of photodynamic therapeutic approaches.

A common strategy for localized photosensitization involves use of chemically modified PS with specific accumulation in organelles or specific cell types. This results in a chemically distinct molecule, typically exposed to cells or organisms at supratherapeutic concentrations, and a distinct photosensitization pathway, where the balance of photosensitized reactive species can vary significantly by compound applied (Zhu et al., 2014). PS–antibody conjugates against tumor antigens are currently in clinical trials for Molecular PhotoImmunoTherapy (Kobayashi and Choyke, 2019). Using a genetically targeted or molecularly targeted strategy enables a localized and active accumulation process, which can enhance PS delivery *via* binding to specific molecules abundant in or on target cells (Giepmans et al., 2006). Genetically targeted PS Killer Red (Bulina et al., 2006), MiniSOG (Ruiz-González et al., 2013), and SuperNova (Takemoto et al., 2013) have all been used to target intracellular organelles, with varying results on efficacy in directed cell killing, depending on the organelle targeted, expression level, and illumination parameters [reviews: (Wojtovich and Foster, 2014; Trewin et al., 2018)]. Targeted and activated photosensitizers (TAPs) using a genetically encoded fluorogen-activating protein (FAP) enable selective photosensitization directed to targeted cellular sites within genetically or molecularly targeted cells (Wang et al., 2017; Ackerman et al., 2019), as recently illustrated in transgenic zebrafish cardiovascular and nervous systems (He et al., 2016; Xie et al., 2020), and subcellular locations including mitochondria and telomeres, among others (Fouquerel et al., 2019; Jang et al., 2019; Qian et al., 2019). Due to the short range of action of ^1^O_2_ in living systems (Kuimova et al., 2009), this targeting and activation of the PS protects adjacent normal cells and reduces damage to nearby tissues, because photoactivity requires coincidence of the dye, the activating protein, and the illumination source (Lovell et al., 2010; He et al., 2016). The intrinsic fluorescence and photochemistry of FAP–TAPs allows fluorescence imaging *in vitro* and *in vivo*, and during photodynamic treatments, it provides optical feedback proportional to the dose of singlet oxygen delivered, a useful feature for tracking PDT dosimetry, recently correlated with improved clinical endpoints (Ong et al., 2020). Improved tissue penetration and photo-ablation efficacy is achieved using PS that activate between 600 and 800 nm, the near-infrared (NIR) spectral range, which has attracted significant attention in PDT methods owing to the availability of some NIR excitable PS (Fowley et al., 2013; Deng et al., 2017). The FAP–TAPs system, with di-iodinated malachite green analogs (MG2I), has optimal excitation at 660–670 nm, and emission in the NIR range at 705 nm, an ideal spectral combination for photosensitization and detection in deep tissues.

Hsieh et al. showed that cells displayed temporally distinct subcellular localization after Photofrin incubation, associated with distinct death phenotypes in response to subsequent photosensitization (Hsieh et al., 2003, 2010). Indeed, the efficiency of photo-induced cell death depends not only on the yield of oxidant species, but more importantly on the specific subcellular site(s) of their origin (Kessel et al., 1997; Oliveira et al., 2011; Bacellar et al., 2015; Kessel and Oleinick, 2018). In some cases, pronounced synergy can be obtained by targeting two subcellular sites simultaneously (Kessel and Reiners, 2017; Martins et al., 2019). There remains a gap in our knowledge of the influence of a specific dose of a particular PS-generated reactive oxygen species, within a specific cell type (Zhu et al., 2014).

Here, we set out to develop a more consistent understanding of the influence of a similar dose of ^1^O_2_ from a common PS in different subcellular locations of the same cell type. Further, we compared the sensitivity to photoinduced cytotoxicity of two different cell types with matched expression location and expression levels, PS concentrations, and light exposure. We build on two aspects of our previous work, which described a genetically encoded fluorogen-activating protein (FAP_dL5^**^_), specifically targeted to various subcellular locations (Telmer et al., 2015), which can bind a heavy atom-substituted fluorogenic dye (MG2I-ester), forming an “on-demand” TAP that selectively produces singlet oxygen and no other reactive oxygen species (He et al., 2016). In this study, to further understand the specific targeting and photo-ablation effects of FAP–TAPs, we generated stable cell lines expressing the FAP in distinct subcellular compartments, selected cells with equal expression levels by fluorescence activated cell sorting (FACS), verified localization and localized activation of the PS dye by fluorescence microscopy, assessed photocytotoxic dose dependence by MTS assay under varied illumination doses, and characterized the cells 24 h after exposure for apoptotic and necrotic cell death by flow cytometry. These studies were conducted in stably transfected HEK-293 cells, with the dL5^**^ FAP located at five distinct cellular locations, including cytoplasm, endoplasmic reticulum lumen, membrane-anchored, mitochondria, and nucleus, constructs previously reported and validated with this FAP and available at Addgene (Telmer et al., 2015) as listed in Table 1.

**Table 1:** Subcellular Locations and Cell Types used, along with Addgene Plasmid Names/Numbers, short names used in this manuscript, and population median fluorescence prior to intensity selection by sorting.

**Cell line/subcellular location**	**Plasmid name (Addgene #)**	**Alternative short name**	**Unsorted median fluorescence**
HEK-G4S-dL5** Cytosol	pcDNA3.1-KozATGdL5-2XG4S-mCer3 (73207)	G4S-dL5**	54,000
HEK-KDEL-dL5** Endoplasmic Retic.	pcDNA3.1-kappamyc-dL5-2XG4SmCer3-KDEL (73209)	dL5**-KDEL	100,000
HEK-TM-dL5** Membrane	pcDNA3.1-kappamyc-dL5-2xG4S-TM (73206)	dL5**-TM	63,000
HEK-COX-dL5** Mitochondria	pcDNA3.1-COXIVCOX8-dL5-2XG4SmCer3 (73208)	Mito-dL5**	38,000
HEK-NLS-dL5** Nucleus	pcDNA3.1-NLS-mycdL5-2xG4S-mCer3 (73205)	NLS-dL5**	42,000
CT26-COX-dL5** Mitochondria	pcDNA3.1-COXIVCOX8-dL5-2XG4SmCer3 (73208)	Mito-dL5**	

## Materials and Methods

### Chemicals and Reagents

We purchased all commercially available chemicals from VWR International or ThermoFisher without further purification. MG2I-ester dye was obtained using the method of our previous work (He et al., 2016). MG-ester dye was obtained using the method of our previous work (Szent-Gyorgyi et al., 2008).

### Cell Culture and Incubation Conditions

All the dL5^**^-expressing HEK293 (HEK) cells, including membrane-anchored FAP, cytoplasmically localized FAP, endoplasmic reticulum-retained FAP, mitochondria-anchored FAP, and nucleus-retained FAP were transfected as pcDNA3.1 plasmids (Table 1) using Lipofectamine 2000 (Invitrogen) and selected by G418, then sorted for stable cell line generation and cultured in fresh DMEM (Dulbecco's Modified Eagle's Medium, Thermo Fisher), containing 1% double resistant (streptomycin and penicillin), 1% G418, and 10% inactivated FBS (fetal bovine serum, FisherBrand) at 37°C under a humidified 5% CO_2_ and 95% air atmosphere. The cells were split before they reached 90% confluence, every 2–3 days.

### Selection of Consistent Expression Levels Across Cell Lines

**Figure 1:**
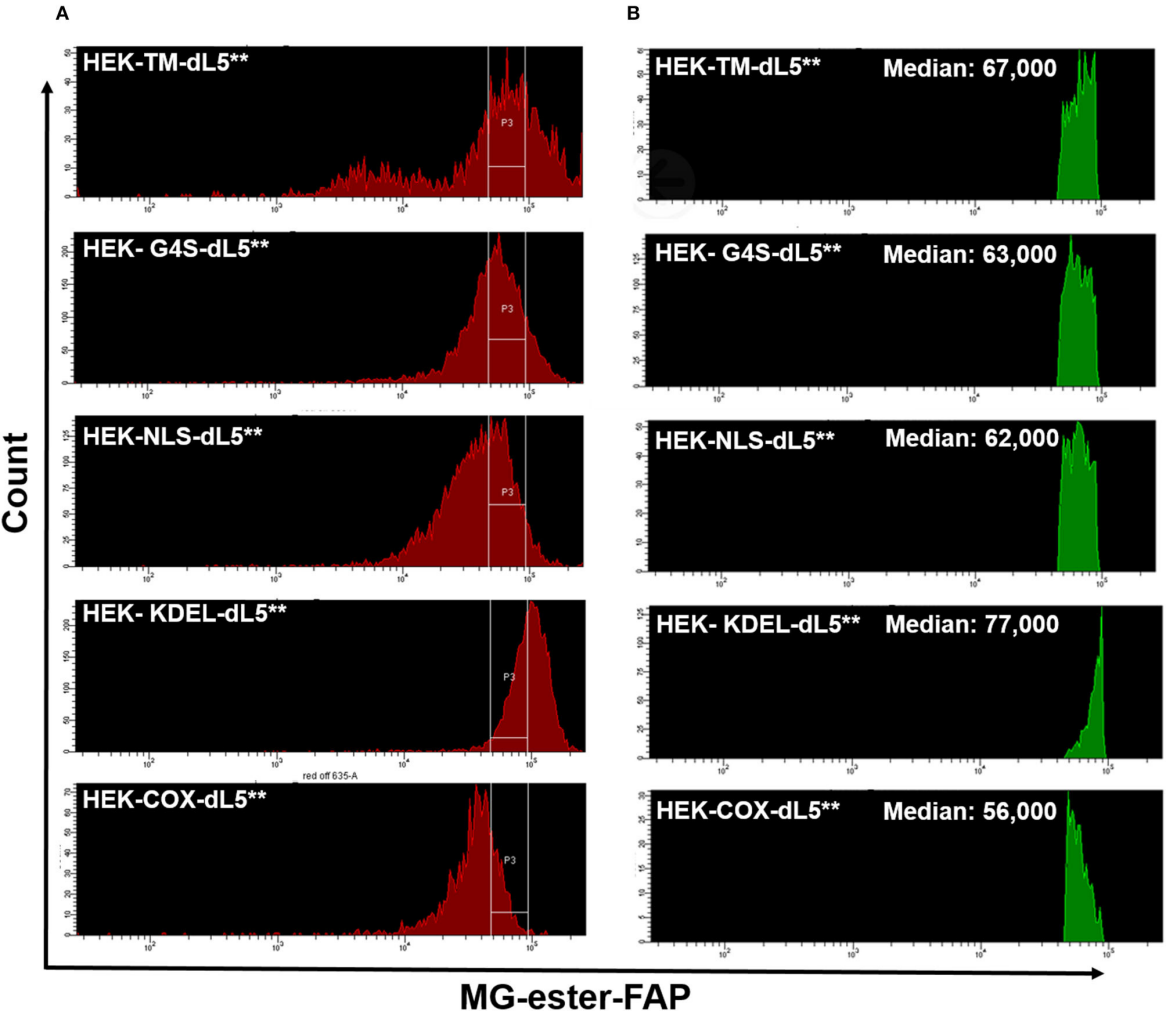
**(A)** Similar expression levels of five different dL5**-expressing HEK stable cell lines prior to sorting (red, left histograms), and the consistent selection gates used to select cells of closely matched expression levels for the experiments (labeled with MG-ester). **(B)** The Green histograms on the right show the MG-ester fluorescence histograms of the population of cells during sorting collection that were within the collection gates P3 indicated for each cell population. The low fluorescence population in the HEK-TM-dL5** cells is a result of lifting the cells using trypsin, which strips plasma membrane protein off some of the cells. Medians of the selected populations were similar, 56,000–77,000, considerably tighter than the >3-fold range of the unsorted median fluorescence populations.

After prolonged selection, expression of FAP constructs in the varied cell lines was typically spread across a 20- to 100-fold range (Figure 1A), as assessed based on fluorescence intensity by flow cytometry. To provide consistent photosensitizing dose, we selected cells using a defined fluorescence intensity gate based on FAP labeling with the MG-ester (a fluorogenic but non-photosensitizing dye that binds to the dL5^**^) (Figure 1B) and expanded them briefly after collection. Subsequent analysis showed that the sorted, selected cells maintained similar expression levels over the duration of the experiment, generally <72 h. Briefly, cells cultured as above were labeled with MG-ester and sorted to collect a population (4 × 10^4^) of cells with emission in a bright and narrow window (median in selection gate is shown in Table 2). The sorted cells were collected using this gate, centered around 70,000 RFU with a width of 44,000 RFU into a culture plate (60 mm). Then, all collected cells were returned to the incubator for future use. Statistics reported in Table 1 were derived using the FACSDiva software. The median of all detected cells and the median of the sorted and the fraction of collected cells were determined by analysis of the ungated and gated populations from the respective cell-sorting procedures.

**Table 2:** Fraction of cells sorted, median fluorescence intensity in selection gate, half-maximal lethal doses (LD50) by MTS assay, and death staining Annexin/PI positive populations for each FAP-localized cell line after illumination.

**FAP** **location** **(*p*-value vs. cytosol)**	**%** **population selected**	**FL4FACS median** **(in gate)**	**95% CI** **LD50** **(J/cm^2^) (95% CI** **Persister fraction)**	**% death** **(FACS** **after 28.8 J/cm^2^)**	**%** **Annexin (+)** **PI (–)**	**%** **Annexin (–)** **PI (+)**
Cytosol (G4S)	46.1	63,000	5.3–8.9	42.0	2.5	39.5
			(0.21–0.34)			
Endoplasmic Reticulum (KDEL, NS)	58.6	77,000	8.1–10.1	42.7	13.1	29.6
			(0–0.05)			
Membrane (TM, *p* = 0.02)	33.5	67,000	6.7–7.3	45.9	28.4	17.5
			(0.03-0.07)			
Mitochondria (COX, *p* < 0.0001)	62.1	56,000	30.3–77.0	15.3	2.1	13.2
			(0-0.35)			
Nucleus (NLS, *p* = 0.0004)	27	62,000	23.7^*^	12.3	2.5	9.8
			(0.34^*^)			

N.D. is not determined, and

* *indicates fit values where confidence intervals could not be obtained from fitting the data with common constraints*.

### Fluorescence Imaging of FAP–TAPs in Different dL5^**^-Expressing HEK Cells

For cell imaging, five different dL5^**^-expressing HEK cell populations were seeded into confocal culture plates (Mattek). After 24 h, the medium was replaced by fresh DMEM with 500 nM MG2I-ester for 3-h incubation in the dark. Then, the cells were rinsed with PBS and wells were refilled with 1 ml of Opti-MEM. Images were captured using a Zeiss LSM 880 confocal (Ex = 633 nm, Em = 700 nm, 64 ×); the same acquisition parameters were used for all images (laser power, detector channel setting, pixel size, and image size). Images displayed are adjusted to a common lookup table to preserve relative fluorescence intensities between the specimens. Larger images are included for each panel in Supplementary Figures 1–5.

### Light Dose-Dependent Cytotoxicity of FAP–TAPs

The five different localized dL5^**^-expressing HEK cell populations were seeded into 96-well white plates at a density of about 1 ×10^5^ cells per well (two plates per cell line) and incubated in a humidified 5% CO_2_ atmosphere for 24 h. The medium was replaced by fresh DMEM, 100 μl/well, with appropriate concentrations of MG2I-ester (treatment group: 500 nM, control group: 0 nM, blank group: 0 nM), and then cells were kept for 3 h in the incubator.

Subsequently, the cells were illuminated for 45 s with a 100-W deep red LED illuminator (emission maximum, 660 nm) using a lens with a 60–80° beam angle to spread the light in a roughly gaussian pattern across the plate, with a peak power of 584 mW/cm^2^. The power density was characterized for each well using Cy5 bleaching as an actinometric calibrant in SBS standard plate format. This arrangement allowed highly reproducible light doses for each well, sampling a >25-fold intensity range across the active plate samples (excluding controls and a 1-well moat perimeter) (Supplementary Figures 6, 7).

After exposure, the medium in the blank group was replaced by 70% ethanol solution for 1 min, sufficient to result in a 0% viability sample value (Tapani et al., 1996). All wells were exchanged to fresh DMEM containing 10% FBS and the cells were returned to the incubator. After 12 h, viability was determined using MTS solution (VWR, 2 mg/ml): 20 μl was added to each well for 4 h. Absorbance intensity was detected at 490 nm using a TECAN M1000 multimode plate reader to determine the MTS reduction extent by the increase in O.D. (optical densities). The “control group” received no MG2I-ester, only brief exposure to 660 nm light, and no photosensitization, thus having maximum viability. Individual well cell viability values were calculated by the formula: Cell viability (Fraction) = [absorbance of well (sample–S) – average absorbance of blank group (EtOH treated–B)]/(average absorbance of control group (Viable–V) – average absorbance of blank group (EtOH treated–B) = (S – B)/(V – B). Viability data were plotted against the determined power at each well position to efficiently generate a photoablation efficacy curve for each cell type and location.

Data were fitted using GraphPad Prism 8.4.3, employing a variable slope normalized dose–response curve to determine absolute IC_50_ light-dose values with the following constraints: baseline: 0; bottom: >0; top: <1.0; IC_50_: <200 J/cm^2^. A fraction of the cell population was not sensitive to increases in light treatment for a given subcellular localized FAP. This “persister fraction” was taken to be the bottom from the fits, and the IC_50_ light-dose values were determined as the IC_50_ values obtained in the fits. Parameters are reported as 95% confidence intervals from the fitting of the individual curves under these constraints. Where the fits could not determine a 95% confidence interval, we reported the value returned from the fit and indicated these data points with an asterisk (^*^). Statistical differences were determined by comparison of each dose–response dataset to the cytoplasmic construct data using a Kruskal-Wallis test for non-normal distributed data, and *p*-values were determined as shown in Table 2.

### FAP–TAPs Induced Apoptosis and Necrosis on Different dL5^**^-Expressing HEK and CT26 Cells

The five different dL5^**^-expressing HEK293 (ATCC CRL1573) and mito-dL5^**^-expressing CT26 (ATCC CRL-2638—a gift from C. Bakkenist) cell lines were seeded into 12-well plates at 10^6^ cells per well (two wells per cell line) and incubated in a humidified 5% CO_2_ atmosphere at 37°C for 24 h in 10% FBS/DMEM media. The medium was replaced by fresh DMEM medium (without FBS) containing MG2I-ester (Control group: COX-dL5^**^ cells, 0 nM, treatment groups: 500 nM). The cells were illuminated for 180 s with 660-nm NIR in a light box using a dispersive, 12-cm distant scattering lens to generate consistent exposure across the plate of 160 mW/cm^2^, similar to our previously published LED illuminators (He et al., 2016; Xie et al., 2020), yielding a total light dose of 28.8 J/cm^2^, followed by addition of medium containing 10% FBS to every well. After incubation for 12 h, the cells were collected and stained with annexin V-FITC (0.4 μg/ml) and propidium iodide (0.5 μg/ml) in binding buffer [0.01 M Hepes (pH 7.4), 0.14 M NaCl, and 2.5 mM CaCl_2_ solution] for 5 min. Cell death percentages were determined for every specimen by flow cytometric analysis of populations increased in PI and/or Annexin V staining relative to control specimens (FITC: Ex = 488 nm; PI: Ex = 535 nm).

## Results and Discussion

### FAP–TAPs Activated Fluorescence Imaging

We set out to characterize the sensitivity of cells to singlet oxygen generation at different subcellular sites, based on our previous work, in which we demonstrated a versatile chemogenetic targeting system that functions in various compartments in mammalian cells (Telmer et al., 2015). We subsequently demonstrated use with our chemoptogenetic heavy atom-substituted fluorogenic dye, forming an “on-demand” TAP that produces ^1^O_2_ at the targeted site within the cell when illuminated with far-red 660-nm light (He et al., 2016). To verify the specific targeting effect of FAP–TAPs, we performed fluorescence microscopy of HEK cell lines expressing different dL5^**^ transgenes specifying distinct subcellular locations for the FAP in the presence of MG2I-ester. These cells were previously validated for organelle-specific targeting using the non-photosensitizing MG-ester dye. The constructs are as listed in Table 1.

**Figure 2:**
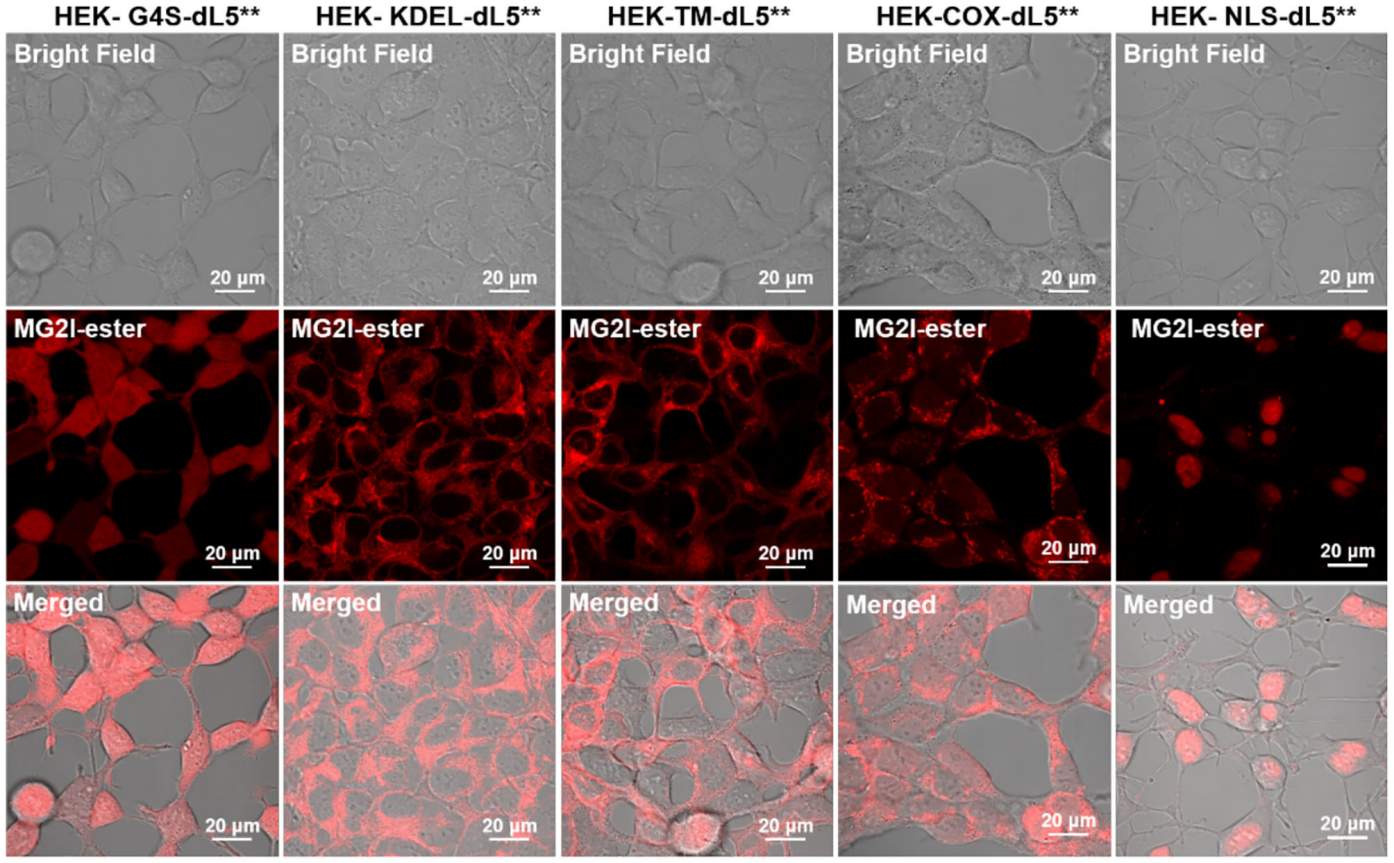
Confocal fluorescence images of five different dL5**-expressing HEK cells incubated with MG2I-ester (500 nM, Ex = 633 nm, 64 ×). These constructs have been validated for localization previously, and the patterns observed in these experiments are consistent with proper localization. For the TM construct, the MG2I-ester, being a cell-permeant fluorogen, labels both the plasma membrane and secretory pathway, including ER, Golgi, and endolysosomal locations. High-resolution images are included as Supplementary Figures 1–5.

As shown in Figure 2 and Supplementary Figures 1–5, each organelle demonstrated its expected localization detected through fluorescence of the bound and activated MG2I-ester dye, demonstrating that the TAPs dye MG2I-ester is activated selectively at the target site within cells, and that cellular generated singlet oxygen will arise from the specified subcellular locations, where the MG2I dye is bound and activated by the localized FAP.

### FAP–TAPs Mediated Cellular Photoablation

**Figure 3:**
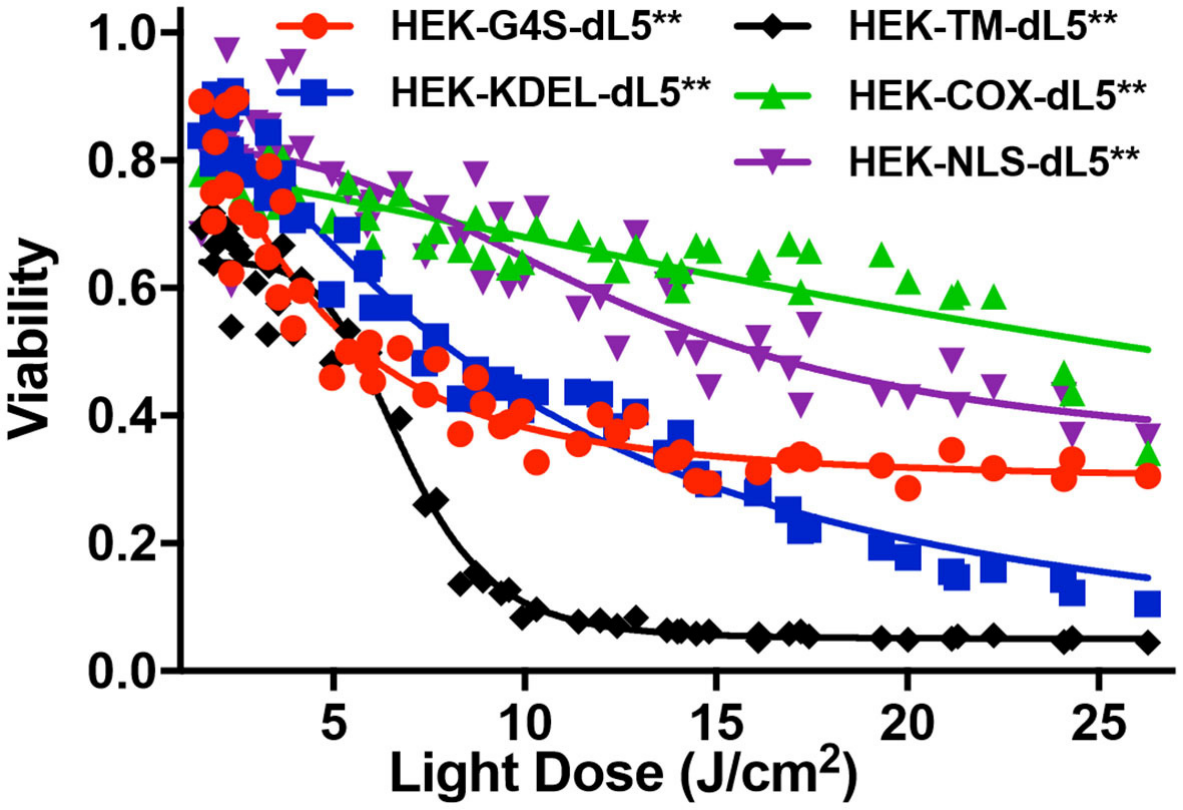
MTS cytotoxic assay of five different dL5**-expressing HEK cells incubated with MG2-Iester (500 nM, NIR 660-nm illumination, 45 s). All plates were treated consistently, with in-plate controls for no photosensitization (no dye: viability = 1), complete cytotoxicity (70% ethanol: viability = 0), and normalized viability plotted for the appropriate photo-exposure of the given well.

To investigate the impact of localized production of singlet oxygen (^1^O_2_) on induction of cell death, we used the MTS cytotoxic assay to characterize the light-induced cytotoxicity of FAP–TAPs on different HEK cells with similar dL5^**^ protein expression levels, targeted to distinct subcellular locations. As shown in Figure 3, the cell viability declined significantly with increasing light dose under NIR 660-nm illumination for all cell lines, with the half-maximal lethal doses (95% confidence intervals of LD_50_) of illumination for distinct locations as in Table 2. Fitted values for LD50 light dose in TAPs-labeled HEK cells with cytoplasmic-, endoplasmic reticulum-, membrane-, mitochondrial-, and nuclear-localized dL5^**^ were 6.9, 8.9, 7.0, 40.5, and 23.7 J/cm^2^, respectively, demonstrating a fivefold difference in light-activated cytotoxicity resulting from similar ^1^O_2_ generation in FAP-expressing HEK cells having distinct subcellular FAP locations. Cells having mitochondrial- and nuclear-localized FAP showed the highest resistance to a locally generated ^1^O_2_ stimulus, possibly owing to their robust maintenance mechanisms, designed to buffer, isolate, and repair specific oxidative lesions, and to the highly localized range of action of the ^1^O_2_ within the cell (Kuimova et al., 2009). Intriguingly, different subcellular locations appear to have different photosensitization-resistant populations, where no further cell death is observed at increasing photosensitization dose. Although potentially interesting and informative (Kessel et al., 1997; Oliveira et al., 2011; Zhu et al., 2014; Kessel, 2019a), a detailed investigation of the mechanisms that determine cell death or survival under these treatments is beyond the scope of this investigation.

### FAP–TAPs Induced Apoptosis and Necrosis

**Figure 4:**
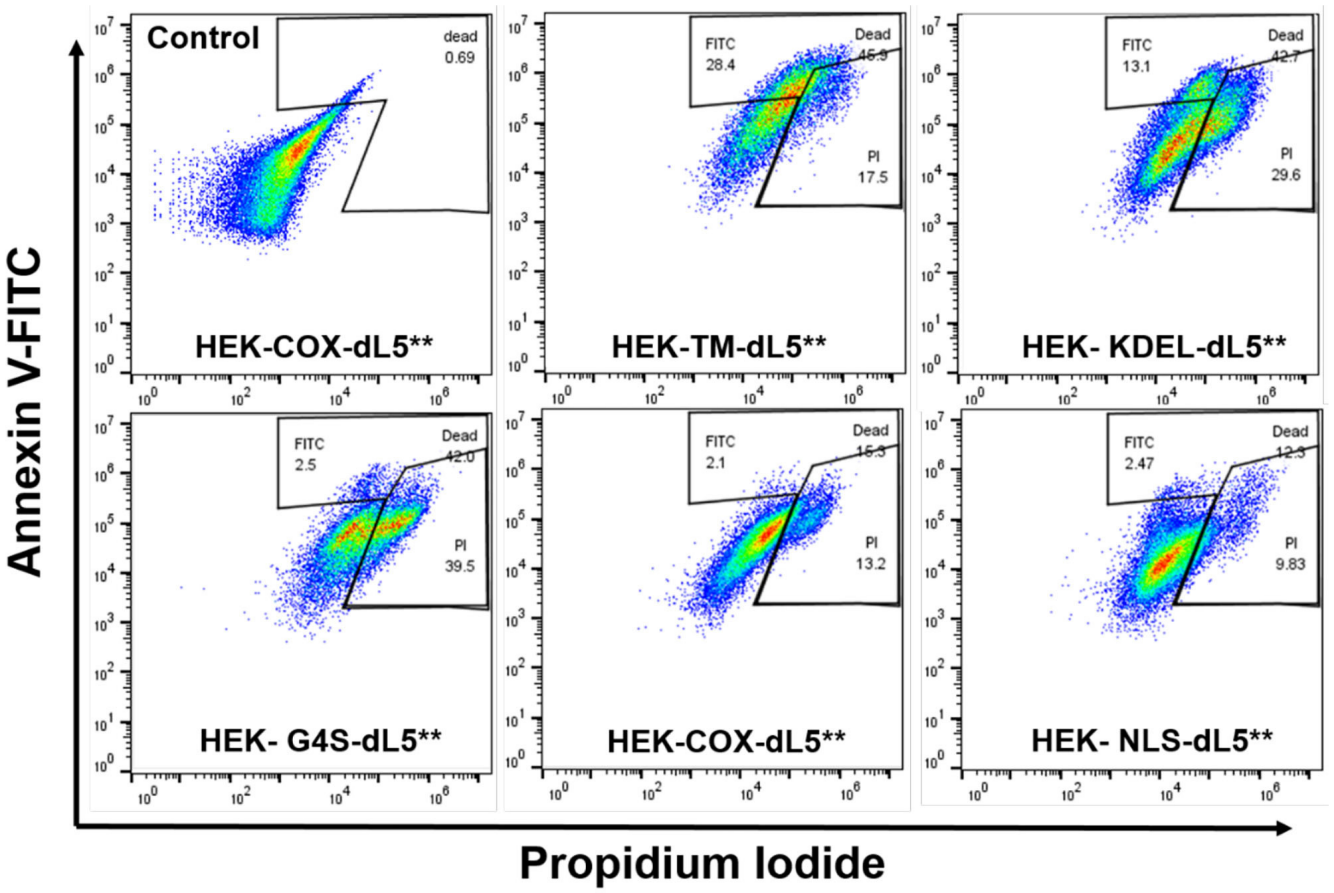
Flow cytometry analysis of five different dL5**-expressing HEK cells incubated with MG2I-ester (500 nM) and exposed to 28.8 J/cm^2^ of 660-nm LED illumination (160 mW/cm^2^, 180 s) (Control HEK-COX-dL5** cells without dye exposure). Different subcellular localization of ^1^O_2_ results in induction of distinct cell death pathways as detected in indicated gate regions with Annexin V-FITC and PI staining, with varied efficacies.

To further characterize the mode of cell death resulting from acute generation of an effective lethal bolus of localized ^1^O_2_, we used a flow cytometric assay with Annexin V-fluorescein isothiocyanate (Annexin V-FITC) and propidium iodide (PI) staining to quantitatively determine the apoptosis and necrosis caused by MG2I-ester in different dL5^**^-expressing HEK cells after NIR 660-nm illumination. As displayed in Figure 4 and Table 2, the total percentage of dead cells in the control group (COX-dL5^**^ without illumination) was just 0.69%, while all treatment groups showed significant levels of total dead/dying cells, thus demonstrating excellent biocompatibility, low dark toxicity, and high phototoxicity of the MG2I-ester-dL5^**^ complex. At the same time point after illumination, the early apoptotic (Annexin+, PI–), necrotic (Annexin–, PI+), and late apoptotic cell (Annexin+, PI+) populations were different for each cellular location.

We found that membrane-restricted singlet oxygen resulted in effective cell ablation, with a predominant population showing a late apoptotic phenotype, having both Annexin V and PI signal enhancement relative to untreated cells. The ER-localized cells displayed two distinct populations, one showing signs of early apoptosis and the other showing predominantly necrosis. The cytoplasmic, mitochondrial, and nuclear cells showed almost exclusively necrotic cell death, with some small populations showing a potentially early apoptotic phenotype in the measured cells. These results, coupled with the similar expression levels among the selected cell lines, establish definitively that the extent and nature of cell death pathways initiated by a similar dose of ^1^O_2_ in HEK cells depend on the subcellular origin. A more detailed understanding of how these differences in origin of reactive oxygen species are recognized and transduced by the cell into distinct cellular responses may reveal important insights into the role of oxidizing modifications in cellular signaling and managing responses to changes in oxidative environments, both natural and interventional.

**Figure 5:**
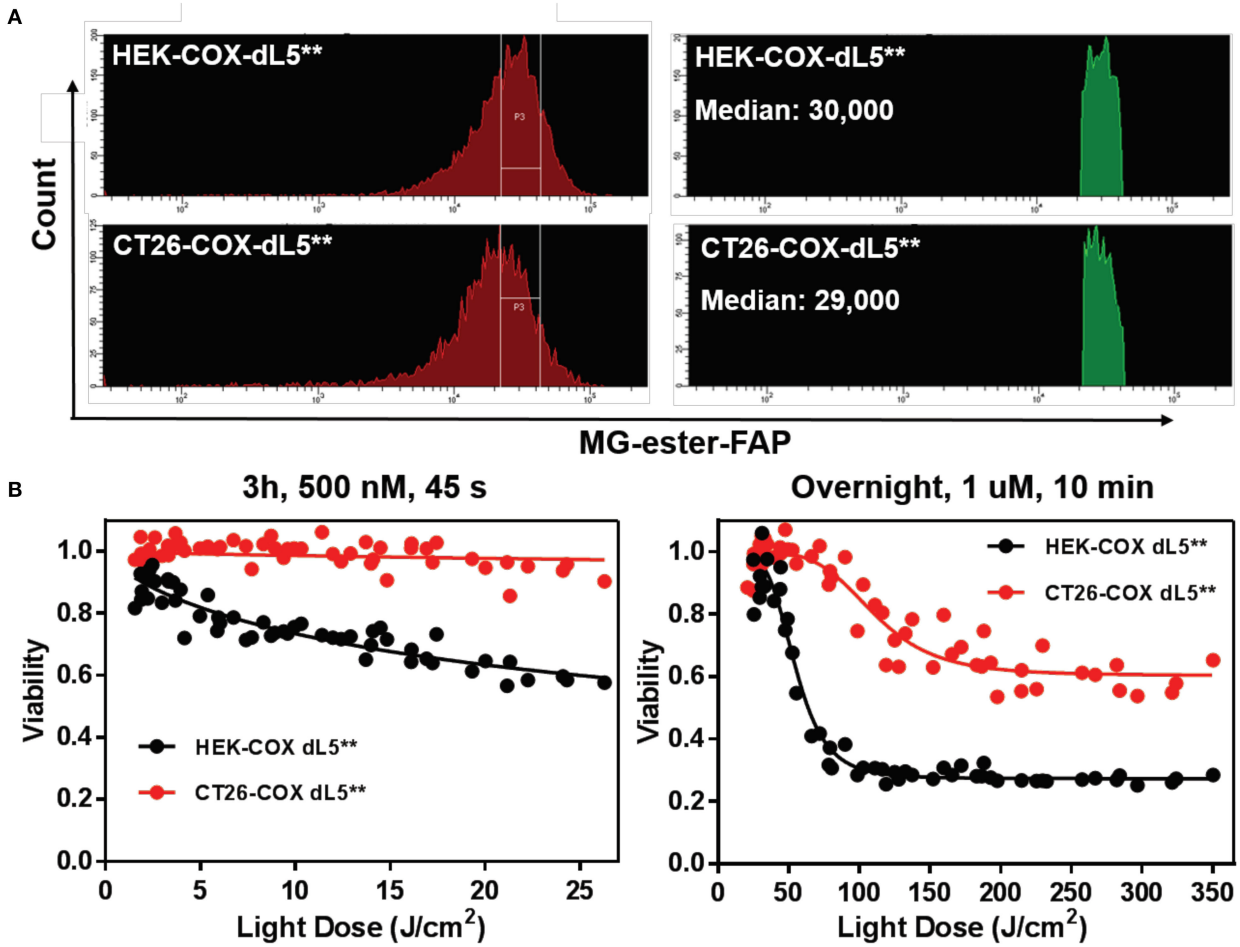
HEK cell and CT26 cells with mitochondrially localized FAP show dramatically different sensitivity to singlet oxygen generation. **(A)** Populations of cells with initially similar FAP expression (MG-ester-FAP signal) were collected within the indicated gate region to narrow the fluorescence distribution, with the population falling in the gate shown as a fluorescence histogram in green on the right, with the associated population medians. **(B)** The phototoxic dose–response curves of collected cells reveal that CT26 cells are remarkably resistant to mitochondrially generated singlet oxygen compared to the HEK cells with similar expression levels.

In order to evaluate the efficacy of reactive oxygen species in mediating cell death in normal and cancer cells, we compared the ablation dose–response curves of HEK cells and CT26 colorectal tumor cells stably expressing the mito-dL5^**^ constructs with similar levels of expression. These cells showed pronounced differences in photodynamic sensitivity, with the tumor cells being far more resistant to the mitochondrially expressed FAP–TAPs singlet oxygen production (Figure 5). Upon exposure to conditions resulting in up to 40% viability reduction in HEK cells, the CT26 cells showed essentially no cytotoxic response. Adjusting the dye exposure and the light dose resulted in increased, but not nearly as effective phototoxicity, with ~60% of the CT26 cells remaining viable regardless of light exposure, while only 28% of the HEK population maintained viability under these conditions. As before, dose-dependent viability data were fitted to a sigmoidal curve with a floor, providing a measure of both the persistent fraction and the midpoint of the photoablation efficacy (IC_50_). The obtained IC_50_ light doses were ~50 J/cm^2^ for HEK cells under these treatment conditions but were >100 J/cm^2^ for the CT26 cells, indicating a strong and significant (*p* < 0.0001, Mann–Whitney test) cell-type-dependent aspect to photodynamic sensitivity, at least for mitochondrial FAPs.

These results outline a clear approach to better understand the mechanism and cellular consequences of directed singlet oxygen-mediated phototoxicity in a variety of relevant cells and oxidative signaling compartments. The genetic encoding of the FAP enables both cellular specificity and subcellular targeting and opens the potential for simultaneously targeting singlet oxygen generation in multiple subcellular locations at the same time, using the same chemical and light exposure. The chemoptogenetic ability to restrict singlet oxygen generation to specific compartments and cells within a complex environment, and to quantitatively activate its generation at a specific time by adding dye and treating with light, enables a range of direct investigations into the role of singlet oxygen in signaling and inducing cell death in a variety of cellular and complex biological contexts (Lan et al., 2014; Fouquerel et al., 2019; Jang et al., 2019; Qian et al., 2019; Binns et al., 2020; Xie et al., 2020).

Other investigators have found both similar and different orders of efficacy in cell ablation when targeting subcellular organelles. Using miniSOG, Ryumina et al. (2013, 2016) found that membrane targeting was more effective than mitochondrial, lysosomal, or nuclear targeting. Xu and Chisolm (Xu and Chisholm, 2016), using miniSOG2, also found membrane targeting more effective than cytoplasmic or mitochondrial targeting. Serebrovskaya et al. (2013) found no significant difference between mitochondrial-, lysosomal-, or membrane-targeted KillerRed, while cytoplasmic KillerRed was ineffective. Wang et al. found peroxisomal and mitochondrial KillerRed more effective than cytoplasmic KillerRed (Wang et al., 2013). On the other hand, Paardekooper et al. found nuclear targeting of SuperNova to give more effective ablation than mitochondrial, endosomal, Golgi, or endoplasmic reticulum targeting (Paardekooper et al., 2019). It is impossible to draw conclusions about which is the most lethal subcellular site, given the different organisms, optogenetic reagents, cell death criteria, and illumination conditions used. Most authors give no data on expression levels at different sites; we have attempted to ensure that the median level of PS expression in cells expressing organelle-targeted FAP–TAPs is similar, although this does not assure that all cells express FAPs uniformly. A further complication is the differing mixes of oxidation products made by different PS (Trewin et al., 2018; Onukwufor et al., 2020). Finally, the efficacy of ablation may depend on the detailed targeting of optogenetic reagents; using either SuperNova (Trewin et al., 2019) or miniSOG (Qi et al., 2012), the precise site targeted in mitochondria determined the results.

The use of ROS-generating PS to analyze signaling pathways is relatively novel but has already produced tantalizing results (Ivashchenko et al., 2011; Shibuya and Tsujimoto, 2012; Wang et al., 2012, 2013; Wojtovich and Foster, 2014; Souslova et al., 2017; Hoffmann et al., 2019; Wojtovich et al., 2019; Berry and Wojtovich, 2020). FAP–TAPs represent a new tool potentially allowing a wide range of new photoactive reagents having multiple activating wavelengths and different ROS generating properties. A strong advantage is that there is no need to minimize light exposure of the target; no photosensitization occurs until the TAP is added. Further advantages include high quantum yields of singlet oxygen generation and far-red absorption, which should allow deep light penetration *in vitro* and *in vivo* and minimize tissue damage.

## Conclusion

In summary, we have shown that a PS, at a consistent concentration per cell, targeted to different subcellular locations and in different cell types produces a variety of cell death responses. These studies were possible due to the unique activation of the PS by the genetically encoded FAP receptor, which allows both targeting and target-site activation of the dye molecule, which can then be stimulated to various extents using controlled light doses. In these studies, we showed that a relatively normal immortalized cell line, the HEK-293 cell line, shows a pronounced sensitivity to membrane-, cytoplasmic-, and ER-associated ^1^O_2_, with mitochondria- and nuclear-localized stimuli showing more limited cytotoxicity and differences in the nominal pathways that mediate cell death. Furthermore, by comparing the sensitivity of a colorectal tumor cell line to these HEK cells, we showed that the tumor cells showed remarkable resistance to photodynamic singlet oxygen generated in the mitochondria. These studies provide useful guidance and new considerations for designing photodynamic therapy. In treatments that require comprehensive cell clearance, it will be prudent to choose PS that localize to subcellular targets or combinations of targets that are most effective at inducing the desired kind of cell death in the target cells. Furthermore, these observations suggest many interesting mechanistic questions that can be answered using this quantitative chemoptogenetic ^1^O_2_ delivery system.

## Data Availability Statement

The raw data supporting the conclusions of this article will be made available by the authors, without undue reservation.

## Author Contributions

PL: experimental design, experiments, analysis, and manuscript preparation. DK: preparation and calibration of the illumination methods and dyes used in studies. YC: experiments and analysis involving cell sorting and flow cytometry. BB: experimental design and manuscript preparation. MB: research design, management, data analysis, interpretation, and manuscript preparation. All authors contributed to the article and approved the submitted version.

## Conflict of Interest

MB is a founder and Chief Technology Officer of Sharp Edge Labs, a company utilizing the FAP fluorogen technology. MB has obtained a patent on the FAP-TAPs technology for targeted cellular ablation, currently owned by Carnegie Mellon University. The remaining authors declare that the research was conducted in the absence of any commercial or financial relationships that could be construed as a potential conflict of interest.
