# Supplementary material for: Subcellular Singlet Oxygen and Cell Death: Location Matters
Source: Front Chem. 2020 Nov 17;8:592941. doi: 10.3389/fchem.2020.592941 (PMC7705227; doi:10.3389/fchem.2020.592941)
Supplement: Supplementary file 1 [file Data_Sheet_1.pdf]

Subcellular Singlet Oxygen and Cell Death: Location Matters

Pingping Liang[1,2,3], Dmytro Kolodieznyi[1,2], Yehuda Creeger[1], Byron Ballou[1], Marcel P. Bruchez[1,2,4*]

[1]Molecular Biosensor and Imaging Center, Carnegie Mellon University, Pittsburgh, PA, USA

[2]Department of Chemistry, Carnegie Mellon University, Pittsburgh, PA, USA

[3]Key Laboratory of Flexible Electronics (KLOFE) & Institute of Advanced Materials (IAM), Nanjing Tech University, Nanjing, China.

[4]Department of Biological Sciences, Carnegie Mellon University, Pittsburgh, PA, USA

*** Correspondence:**
Marcel P. Bruchez
bruchez@andrew.cmu.edu

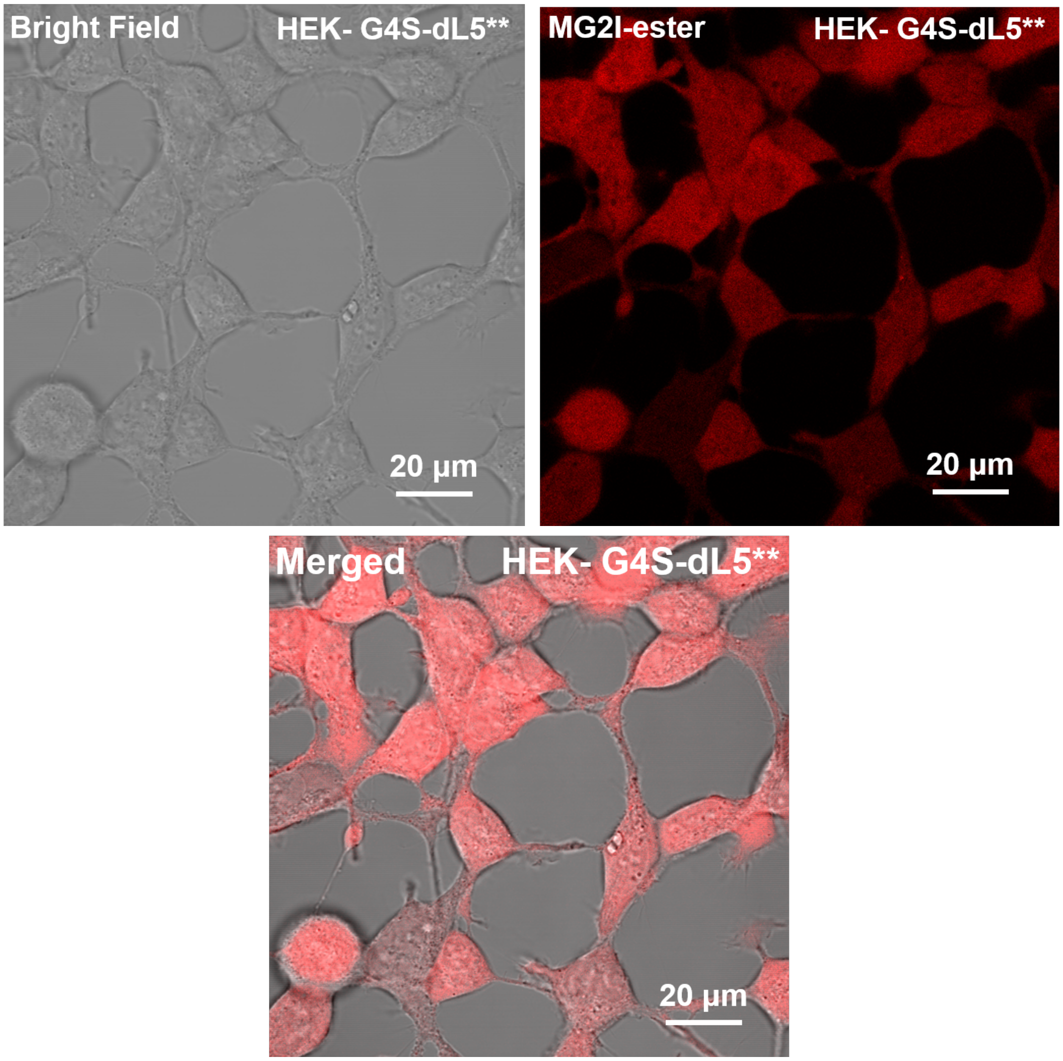

Supplemental Figure 1. High resolution of confocal fluorescence image of HEK-G4S-dL5** cells incubated with MG-2I-ester (500 nM, Ex = 633 nm, 64 ×) Top, DIC image, Confocal fluorescence only, bottom, DIC with overlayed Confocal Fluorescence.

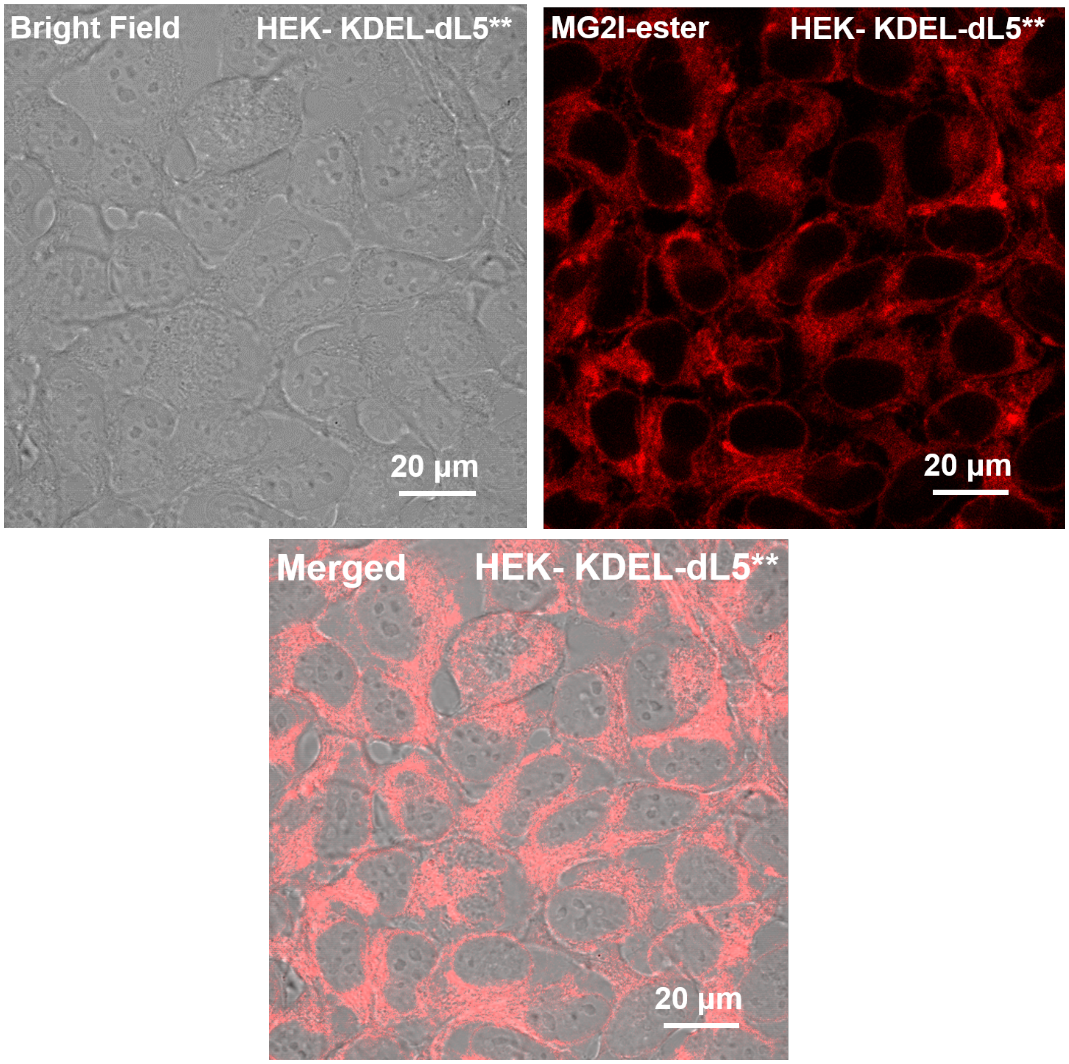

Supplemental Figure 2. High resolution confocal fluorescence image of HEK-KDEL-dL5** cells incubated with MG-2I-ester (500 nM, Ex = 633 nm, 64 ×).Top, DIC and Confocal Fluorescence only, Bottom, DIC overlayed with Confocal Fluorescence.

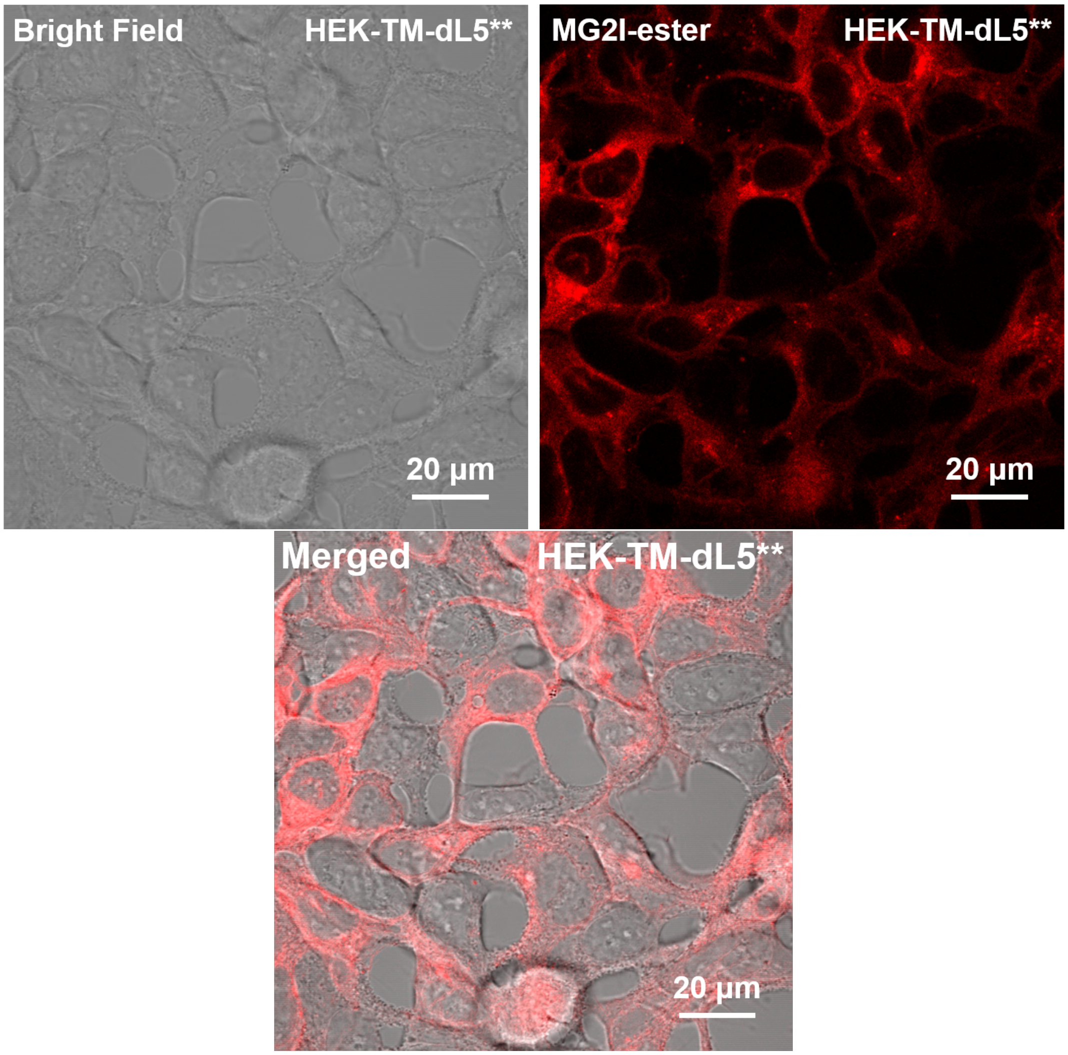

Supplemental Figure 3. High resolution of confocal fluorescence image of HEK-TM-dL5** cells incubated with MG-2I-ester (500 nM, Ex = 633 nm, 64 ×). Top, DIC and Confocal Fluorescence only, Bottom, DIC overlayed with Confocal Fluorescence.

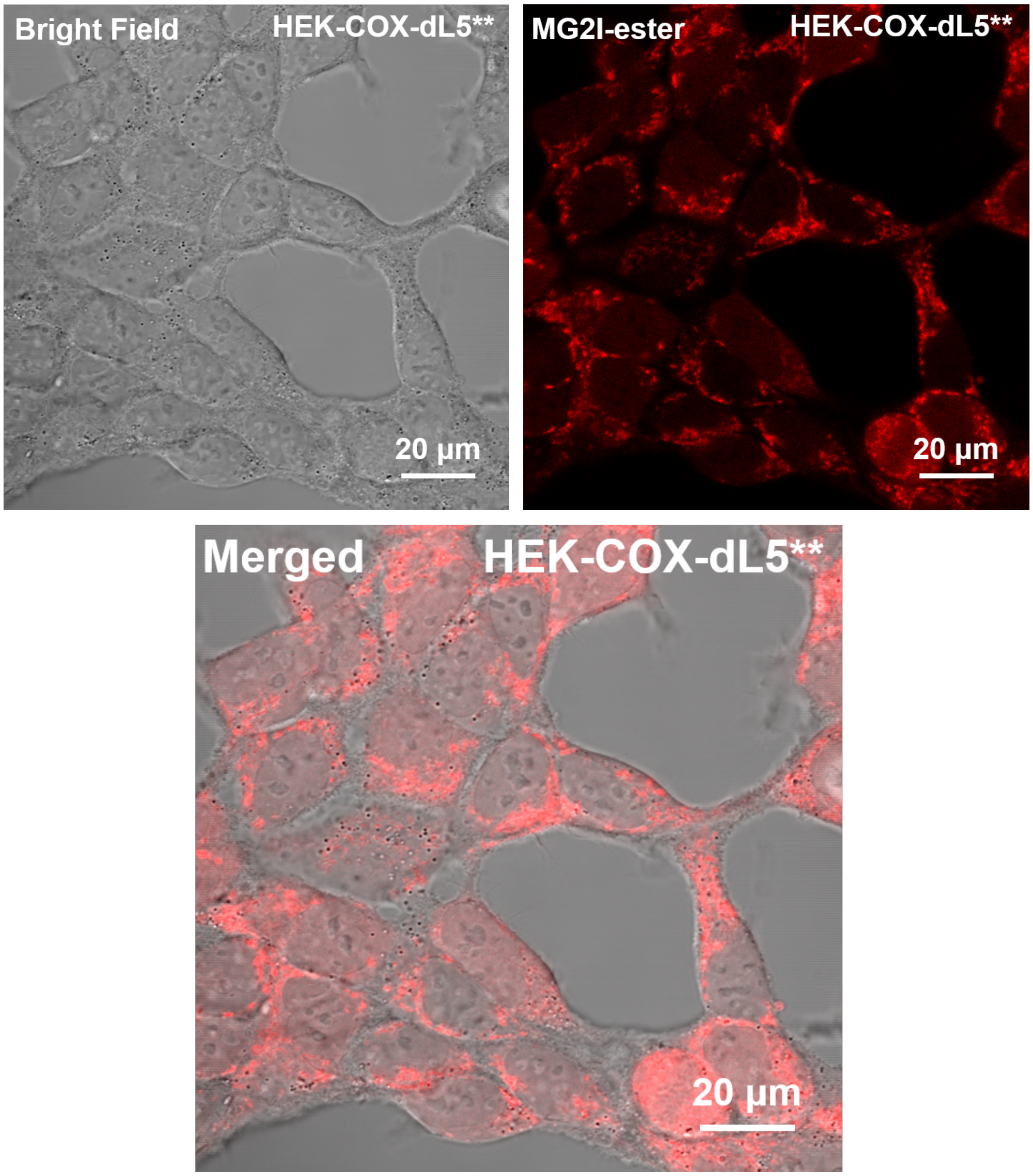

Supplemental Figure 4. High resolution of confocal fluorescence image of HEK-COX-dL5** cells incubated with MG-2I-ester (500 nM, Ex = 633 nm, 64 ×). Top, DIC and Confocal Fluorescence only, Bottom, DIC overlayed with Confocal Fluorescence.

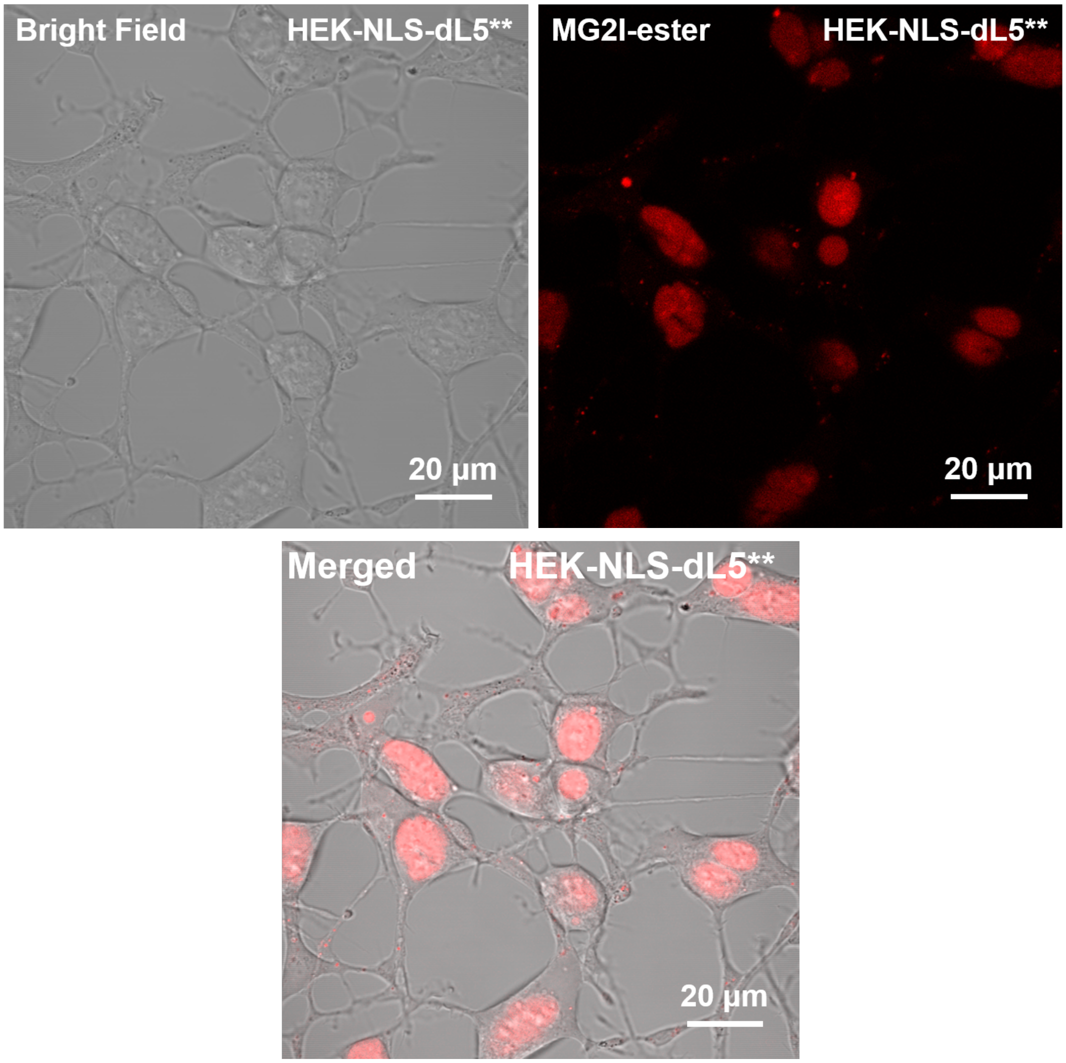

Supplemental Figure 5. High resolution of confocal fluorescence image of HEK-NLS-dL5** cells incubated with MG-2I-ester (500 nM, Ex = 633 nm, 64 ×). Top, DIC and Confocal Fluorescence only, Bottom, DIC overlayed with Confocal Fluorescence.

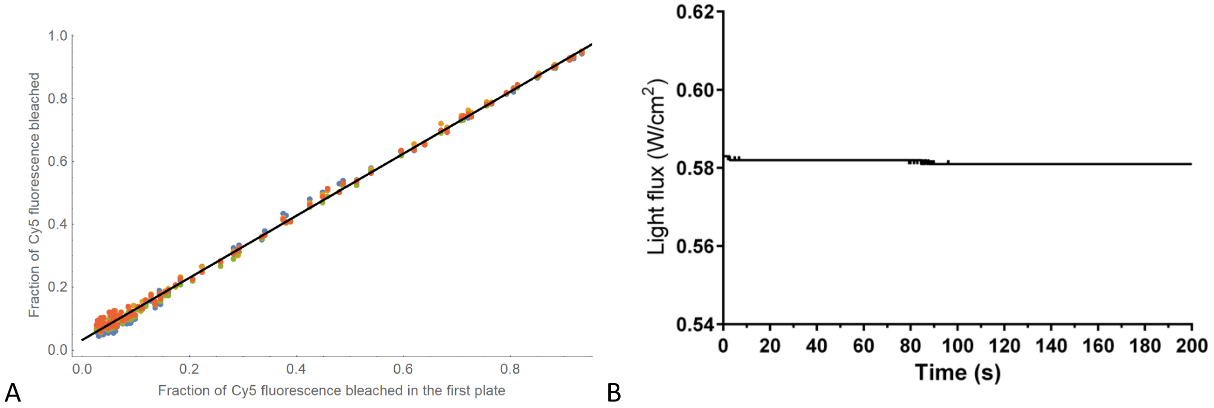

Supplemental Figure 6. Reproducibility of light exposure across multiple plates in the dispersive light box. **A.** To verify the reproducibility of the bleaching between several exposures we tested bleaching of five replicates of plates filled with Cy5. Data is plotted as paired points of individual wells comparing the first plate to subsequent plates. Experimental slope is 0.989 (R2 for fitting is 0.998) indicating the light-dose per well is highly consistent across multiple plate experiments. **B.** Stability of the light flux from the LED was checked by continuous reading of the light power from the LED at the height of the sample using Coherent FieldMaxII-TOP light meter with OP-2 VIS light sensor and a 1000:1 attenuator. During the illumination time used for experiments in this paper, light output from the LED was stable, with variation <1% in intensity.

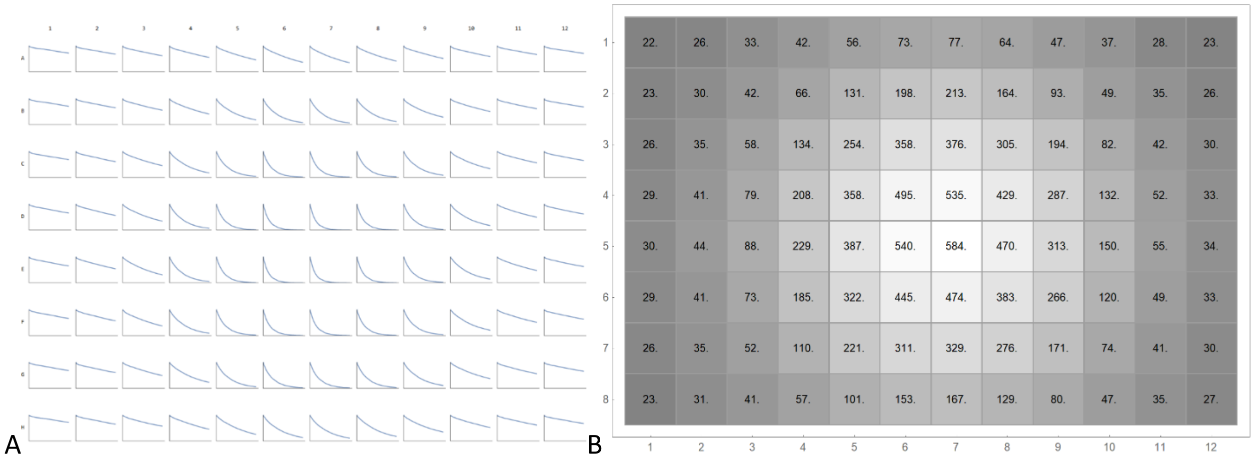

Supplementary Figure 7. Quantitative calibration of light-dose per well in the dispersive LED illuminator. **A.** To evaluate light dose delivered to each well, a 96-well plate with 100 µL of 10 µM Cy5.18 solution dispensed into each well was illuminated for a series of time-points and the fraction of the remaining fluorescence was measured after each time point. Resulting curves for each of the wells were fitted using monoexponential decay and the ratio of the exponent factors was used as a ratio between the light flux that each well is receiving, relative to the measured power at the center of the illumination pattern. This is a valid approach since all other factors (such as extinction coefficient, bleaching rate, etc.) are the same for each well. **B.** The calculated power density at each well using this bleaching-rate analysis to derive per-well illumination powers (in mW/cm^2).
